# Lumbar spine MRI in upright position for diagnosing acute and chronic low back pain: statistical analysis of morphological changes

**DOI:** 10.1007/s10195-012-0213-z

**Published:** 2012-09-16

**Authors:** Umberto Tarantino, Ezio Fanucci, Riccardo Iundusi, Monica Celi, Simone Altobelli, Elena Gasbarra, Giovanni Simonetti, Guglielmo Manenti

**Affiliations:** 1 Department of Orthopedics and Traumatology, “Tor Vergata” University of Rome, “Policlinico Tor Vergata” Foundation, V.le Oxford 81, 00133 Rome, Italy; 2 Department of Diagnostic Imaging and Interventional Radiology, Molecular Imaging and Radiotherapy, “Tor Vergata” University of Rome “Policlinico Tor Vergata” Foundation, V.le Oxford 81, 00133 Rome, Italy

**Keywords:** Upright MRI, Novel diagnostic tool, Low back pain, Lumbar instability, Disc degeneration

## Abstract

**Background:**

Patients with low back pain frequently demonstrate recumbent magnetic resonance imaging (MRI) alterations not always related to homogeneous clinical symptoms. The purpose of this study was to evaluate and quantify the statistical significance of variations of some anatomical parameters of the lumbosacral spine and reveal occult disc pathologies from recumbent to upright position in patients with acute and chronic low back pain.

**Materials and methods:**

Fifty-seven patients complaining of low back pain (27 women, 30 men) underwent dynamic lumbosacral MRI with a 0.25-T tilting system (G-scan Esaote). We settled five parameters for which variations have been evaluated: lumbosacral angle, lordosis angle, L3–L4 intersomatic disc height, L3–L4 interspinous processes distance, and widest anteroposterior dural sac diameter. Images were obtained in both recumbent and upright positions.

**Results:**

Statistically significant differences [one-way analysis of variance (ANOVA), *p* = 0.0043] were found between each pair of values of parameters sampled in recumbent and upright positions. In 70 % of patients, on visual qualitative analysis only, an increment of disc protrusions and/or spondylolisthesis was found in the upright position; in three cases, in the upright position only, an interarticular pseudocyst was found.

**Conclusions:**

Dynamic MRI with an open-configuration, low-field tilting MRI system is a feasible and promising tool to study degenerative pathology of the spine. Moreover, in cases of low back pain with negative MRI in the recumbent position or in patients with pain in the upright position only, tilting MRI permits visualization of occult spine and disc pathologies in patients with acute or chronic low back pain.

## Introduction

The lumbosacral spine is a complex biomechanical system that can adapt to various stresses to which it is subjected: the physiological load and various mechanical stresses produced by posture, daily activities, and traumatic events can accelerate its aging process. Degenerative disease of the lumbosacral spine is therefore one of the most common causes of disability. In this instance, degenerative disease is actually a generic term encompassing a wide range of different disease processes ranging from herniated discs to the pathology of yellow ligaments [1, 2]. Low back pain is an extremely frequent disease that most people experience at some point in their lives; estimates of the 1-year incidence of a first-ever episode of low back pain range between 6.3 % and 15.4 %, whereas estimates of the 1-year incidence of any episode of low back pain range between 1.5 % and 36 % [3]. The imaging techniques using traditional magnetic resonance imaging (MRI) have the major limitation of studying the spine in a position of relative functional rest, as images are acquired with the patient in the supine position while the pain exacerbates in the upright position. False negatives in MRI of the spine performed in the supine position are often due to patient position, with knees and hips bent and spinal variation with increasing breadth of the foramen and vertebral canal. Pathological conditions underlying clinical symptoms, often prompted by standing or sitting, are therefore not seen [4, 5]. This can result in negative findings, even in the presence of symptoms, or an underestimation of pathological specimens. Regardless, the final result is distorted.

Until a few years ago, X-ray was the only practicable imaging modality for the spine in the upright position. This examination is valid and useful for evaluating spinal curvatures, but it shows its limitations when the assessment should be directed to disc structures or when it is necessary to obtain measurements free from problems due to overlapping of anatomical images. A first attempt to evaluate the spine under the loading condition was done with the axial load technique, which is to simulate physiological loading of the spine in the orthostatic position. Although results were certainly interesting, the technique has not achieved a general consensus. Studies with axial load, even if they allow better assessment in relation to the higher signal-to-noise ratio (SNR) afforded by the high-field equipment, do not allow evaluation of the influence that physiological load—represented by the weight of the head and body and by muscle activation—has on the lumbar spine, simulating a load with caudate–cranial direction [6–8].

The technological advancement of open equipment with low- and medium-intensity magnetic field, greater gradient homogeneity, and faster sequences resulted in a significant improvement in SNR in spatial and contrast resolution and therefore image quality. Some MRI equipment is capable of obtaining images of the spine in orthostatic position, which should better evidence pathological conditions that are sometimes “invisible” in the supine position. These devices, all characterized by being open, also have the advantage of eliminating the patient’s feeling of claustrophobia, which sometimes limits diagnostic evaluation of the spine [9–12].

Some publications In the literature involve studying the lumbar spine using MRI equipment with the patient in the upright position, many with medium resistive magnetic field (0.6 T); often, however, observation of physiological and pathological changes detected is not accompanied by a full statistical analysis, which in a certain way, verify the findings observed [13–16]. The purpose of this paper is to provide a statistical evaluation of the variations observed in physiological and pathological parameters of the lumbosacral spine in patients with acute and chronic low back pain studied with new low-field MRI equipment in supine and upright positions.

## Materials and methods

Inclusion criterion was lumbar back pain experienced in standing position. Exclusion criteria were previous spine surgery at any level and/or referred inability to maintain standing position for the scheduled examination time. Fifty-seven patients [27 women (47 %) and 30 men (53 %); mean age 48 (±15) years (women 51 years, men 46 years)], each with a history of low back pain, were studied. Based on the time of painful symptom onset, patients were divided into two groups: acute (within 90 days) and chronic (>90 days) of onset. Among women, ten of 27 (37 %) showed acute onset and 17 of 27 (63 %) chronic onset; among men 11 of 30 (37 %) had acute pain and 19 of 30 (63 %) chronic pain. Fifty percent of patients reported symptoms in the supine position, whereas they all reported pain in the standing position: 34 patients (60 %) had never undergone any diagnostic procedure or had a negative MRI in the supine position; 23 (40 %) had a positive diagnosis for spinal disorder with other diagnostic tools (X-rays, MRI, CT). All patients underwent lumbosacral spine MRI in supine and upright positions, and all gave informed consent prior to being included in the study; local ethics committee authorization was not required because of the standard of care. This investigation was performed in accordance with the ethical standards of the 1964 Declaration of Helsinky as revised in 2000.

The machine used is a permanent magnet of 0.25 T (Esaote G-SCAN), which allows spinal imaging in both supine and upright positions using a tilting system that can rotate from 0° to 90° without the need for patient repositioning. The study was carried out at a table angle of 82° in the upright position, as described in the literature [1], biomechanically reproducing an orthostatic position without incurring patient stability problems, as can be verified by tilting the table to a 90° angle.

With the patient in the supine decubitus, survey sequences were acquired. Sequences were as follows: fast spin echo (FSE) sagittal T2-weighted (TR 3,460 ms, TE 120 ms, 224 × 208 matrix, FOV 320 × 320 mm, mean of three samples; 12.slices 4-mm thick with 0.5-mm gap, acquisition time 5 min 39 s); SE sagittal T1-weighted (TR 580 ms, TE 22 ms, matrix 224 × 208, FOV 300 × 300 mm, mean of two samples; 12 slices 4 mm thick with 0.5 mm gap, acquisition time 4 min 54 s); FSE T2-weighted in the oblique axial plane over intersomatic spaces L3–L4, L4–L5, and L5–S1 (TR 3,880 ms, TE 120 ms, 192 × 192 matrix, FOV 260 × 260 mm, average over three samples, 12 slices 4-mm thick with 0.5-mm gap, acquisition time 6 min 20s ). Then the complex subject-magnet was rotated vertically to 82°. Sequences obtained in this configuration are as follows: sagittal FSE T2-weighted (TR 3,460 ms, TE 120 ms, 240 × 240 matrix, FOV 320 × 320 mm, mean of three samples, 12 slices 4.0-mm thick, 0.5-mm gap, acquisition time 5 min 39 s); FSE T2-weighted in the oblique axial plane over intersomatic spaces L3–L4, L4–L5, and L5–S1 (TR 3,880 ms, TE 120 ms, matrix 192 × 192, FOV 260 × 260 mm, mean of three samples, 12 slices 4-mm thick with 0.5 mm gap, acquisition time 6 min 20 s). It was not possible to follow the same order of sequence acquisitions for all patients. In cases of severe low back pain, we preferred to acquire the first sequences in the upright position to minimize any motion artefacts related to the position, and followed by the supine position, which itself is a less challenging position for these patients.

### Image analysis

To evaluate and quantify anatomical and pathological changes identified in the study performed in both supine and standing positions, the following parameters were taken into account:

1. Lumbosacral angle: This was defined as the anterior open-angle intercepted by two tangent lines of the anterior walls of L5 and S1 (Fig. 1a, b). The normal range for this angle is 120–180°. An increased angle corresponds to vertical tilting of the sacrum, which biomechanically produces an increased load on the anterior column and accelerates the degenerative processes of the L5–S1 disc. On the contrary, a decreased lumbosacral angle is associated with sacrum horizontalization, which consequently creates an amplified load on the posterior elements (facet joints).

**Fig. 1:**
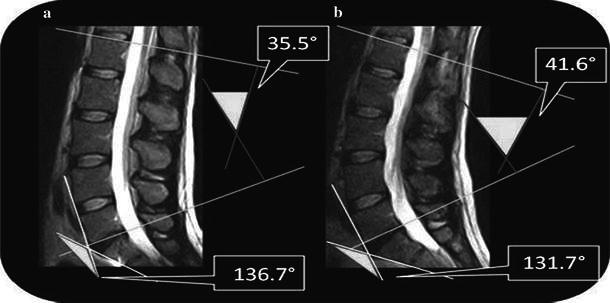
Fast spin echo (FSE) T2-weighted magnetic resonance images (MRI) in the sagittal plane. Lumbosacral angle and lumbar lordosis angle are average values. **a** Supine position: lumbosacral angle 136.7°, lordosis angle 35.5°. **b** Upright position: lumbosacral angle 131.7°, lordosis angle 41.6°
2. Lordosis angle: This was defined as the superior open-angle intercepted between the two perpendicular lines to the tangent of the superior endplate of L1 and the inferior endplate of L5 (Fig. 1a, b). This angle has a normal value of about 50°.
3. Disc height: This is measured at the point of maximum distance between the inferior and superior endplates of L3 and L4, respectively (Fig. 2a, b).

**Fig. 2:**
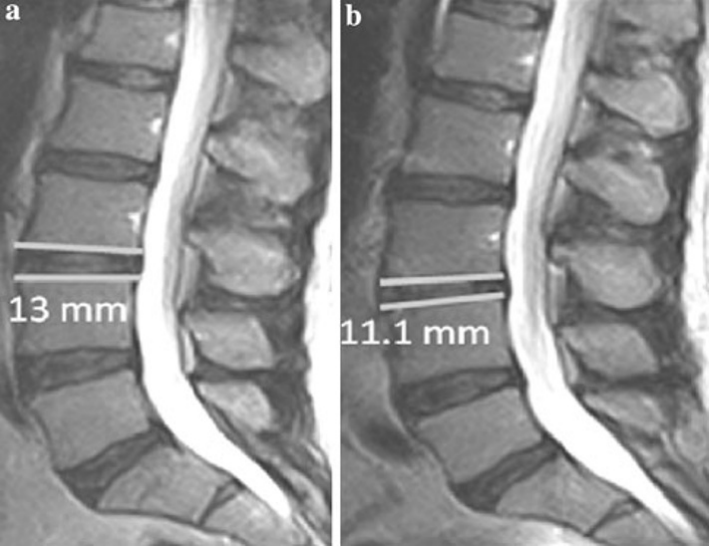
Fast spin echo (FSE) T2-weighted magnetic resonance images (MRI) in the sagittal plane. Assessment of changes in thickness of the intervertebral disc in the transition from **a** supine to **b** upright position, with averaged values
4. Interspinous distance between L3 and L4 (Fig. 3a, b).

**Fig. 3:**
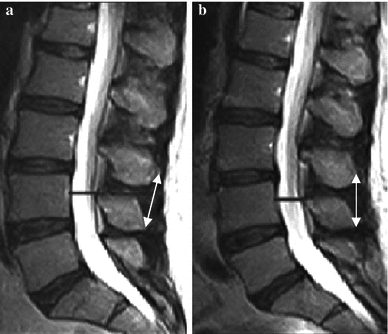
Fast spin echo (FSE) T2-weighted magnetic resonance images (MRI) in the sagittal plane. Assessment of changes in interspinous distance (*white arrows*) and amplitude of the dural sac (*black bars*) in the transition from **a** clinostatism to **b** orthostasis
5. Maximum anteroposterior diameter of the dural sac (Fig. 3a, b).

Morphovolumetric differences in disc protrusions, herniations, and spondylolisthesis with the patient in the supine and standing positions have not been quantified in terms of statistical variations in this paper; they are the focus of a further investigations.

### Statistical analysis

Data collected from measurements taken in supine and standing positions are reported as mean and relative standard deviation (SD); therefore, comparison tests were performed with parametric statistics [*t* test, one-way analysis of variance (ANOVA)]. In this analysis ANOVA is the most useful test because it allows evaluation of the effect due to gender (male–female) with normalized age variables.

## Results

**Fig. 4:**
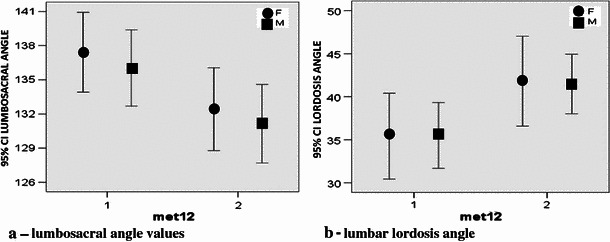
Statistical distribution: Changes in **a** lumbosacral and **b** lordosis angle. **a** Clinostatism: lumbosacral 136.7°, lordosis 35.5°. **b** Orthostasis: lumbosacral 131.7°, lordosis 41.6°

The study was performed on 53 of the 57 patients recruited; four individuals presented with acute symptoms, and study in upright position was saddled with motion artifacts. For the measured parameters, detected findings are described below:

Under physiological conditions, in the transition from supine to upright position, there was a decrease in the lumbosacral angle and an increase of lordosis angle. In supine position, lumbosacral angle had a mean value of 136.7° (women 137.4° ± 8.3°; men 136.0° ± 8.8°) and in standing position 131.7° (women 132.4° ± 8.6°; men 131.1° ± 9.1°) (ANOVA, *p* = 0.00089); No statistically significant difference between sexes were found (Figs. 1a, b, 4a).

**Fig. 5:**
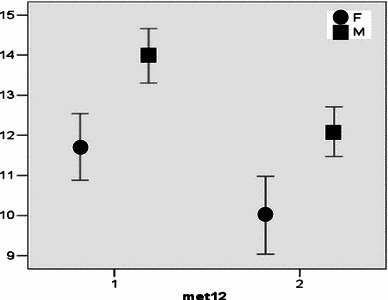
Statistical distribution of the intersomatic disc thickness between sexes. Average value in clinostatism 12.9 mm and orthostasis 11.2 mm

In the supine position, lordosis angle had a mean value of 35.5° (women 35.4° ± 11.8°; men 35.5° ± 10°) and in upright position 41.6° (women 41.8° ± 12.3°; men 41.5° ± 9.1°) (ANOVA, *p* = 0.00097). No statistically significant difference between sexes was found (Figs. 1a, b, 4b). As previously described, intervertebral disc thickness was reduced from supine to standing position, with a mean of 12.9 mm (women 11.7 ± 2 mm; men 14.0 ± 1.8 mm) and 11.2 (women 10.0 ± 2.3 mm; men 12.1 ± 1.6 mm), respectively (ANOVA, *p* = 0.000083). There was a statistically significant difference between sexes (Figs. 2a, b, 5).

**Fig. 6:**
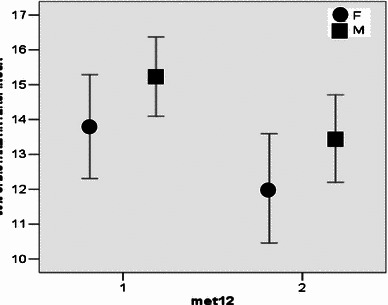
Statistical analysis of variation of the interspinous distance between sexes. Average value in clinostatism 14.6 mm and orthostasis 12.8 mm

The distance between the spinous processes at L3–L4 presented significant differences: mean 14.6 mm (women 13.8 ± 3.5 mm; men 15.2 ± 2.9 mm) and 12.8 mm (women 12.2 ± 3.7 mm; men 13.5 ± 3.3 mm), respectively, in supine and standing positions (ANOVA, *p* = 0.0073). There was a significant difference between sexes in interspinous distance, with men greater than women (ANOVA, *p* = 0.0039) (Figs. 3a, b, 6).

**Fig. 7:**
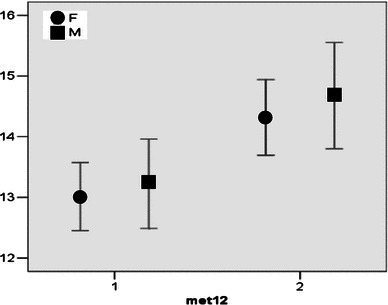
Statistical analysis of amplitude variation of the dural sac between sexes. Average value in clinostatism 13.1 mm and orthostasis 14.5 mm

Anteroposterior diameter of the dural sac in supine position was a mean of 13.1 mm (women 13.0 ± 1.3 mm; men 13.2 ± 1.9 mm) and 14.5 mm in upright position (women 14.3 ± 1.5 mm; men 14.7 ± 2.3 mm) (ANOVA, *p* = 0.00068), without significant differences between sexes (Figs. 3a, b, 7).

**Fig. 8:**
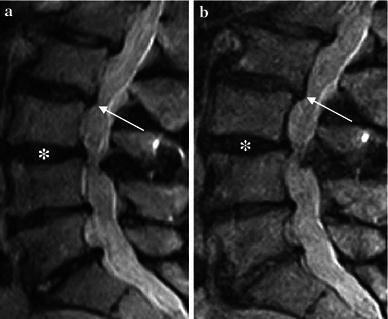
Fast spin echo (FSE) T2-weighted magnetic resonance images (MRI) in the sagittal plane. Disc protrusions in the entire L1–S1 section: **a** Supine, **b** standing. Slight accentuation of the protruding component in the upright position between L2 and L3 (*arrows*) and reduced discal height in the interspace between L3 and L4 (*asterisks*) is visible

**Fig. 9:**
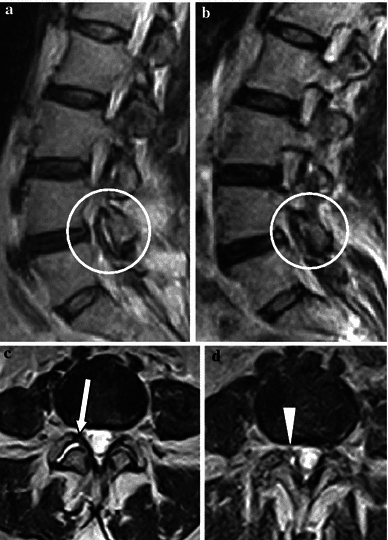
Fast spin echo (FSE) T2-weighted magnetic resonance images (MRI) in the sagittal and axial planes. **a**, **c** Clinostatism; **b**, **d** orthostasis. **c** Presence of a fluid collection between articular facets at L4–L5 (*arrow*). **d** Orthostatic position shows evagination of pseudocystic appearAnce of the right joint capsule with an impression on the nerve root and dural sac (*arrowhead*)

Pathological changes were found in all patients. The most common findings were disc protrusions (44), disc herniations (12), facet-joint pathologies (10), spondylolisthesis (4), spinal canal stenosis (1), and pseudocysts of the joint capsules (3). Upright MRI showed a significant volumetric increase of disc protrusions than standard MRI. Moreover, upright MRI demonstrated disc protrusions in 11 patients with negative findings in supine position (Fig. 8a, b). In one case, a pseudocyst of the facet joint leading to a compressive effect on the nerve root was found in the standing position only (Fig. 9a–d).

**Fig. 10:**
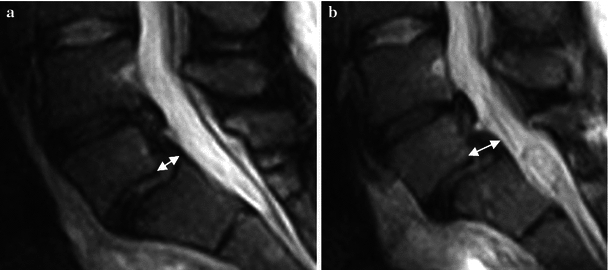
Fast spin echo (FSE) T2-weighted magnetic resonance images (MRI) in the sagittal plane. **a** Clinostatism; **b** orthostasis. Listhesis is accentuated in the upright position passing from grade I to II and making disc protrusion more evident

Four cases of spondylolisthesis, observed in supine position, illustrated aggravation in upright position during the same study (Fig. 10a, b).

## Discussion

The evaluations of physiological and biomechanical elements showed that for each considered parameter, there are significant and meaningful differences depending on supine or upright position and sometimes even on gender; careful analysis of an MRI should therefore be performed according to these data when interpreting subsequent pathological findings. Reduced lumbosacral and increased lordosis angle depend on activation of postural effects of body weight mediated by abdominal and paraspinal muscles. In standing position, the lumbosacral angle decreases in relation to verticality of the spine, which is necessary to support the increase in weight, whereas the increase in lordosis angle reflects compensation by muscle contracture. The reduction of intervertebral disc height was highly significant (*p* = 0.000083), undoubtedly due to body weight and muscle activation [11, 14, 17]. In particular, the reduction of disc height affects the posterior portion, whereas anteriorly, there was a slight increase, with major changes at L2–L3 and L3–L4 [18].

Observed variations in the lordosis and lumbosacral angle values confirm the already known physiological changes produced by the transition from supine to orthostatic position. From a biomechanical point of view, reduced intervertebral disc thickness is closely related: the disk is the fulcrum of a lever in which the resistance is represented by facet joints, and muscles are the power. These aspects emphasize the high sensitivity of the method in evaluating changes in physiopathological discs. These aspects confirms the high sensitivity in assessing the lumbar spine under load conditions, thereby putting some stress on the importance of upright MRI in all those cases where the supine MRI assessment is negative.

Another parameter with a high statistical significance with regard to variations was the anteroposterior diameter of the dural sac. This aspect was previously assessed [11, 19], but in terms of spinal canal area and a reduction of 5.2 % of the dural sac, which was evident in the transition from the supine to the upright position, relating to two variables: change in position of disc and yellow ligaments. These factors undoubtedly influenced canal measurements. Width reduction of the canal was also closely related to the possible redundancy, in the upright position, of the meninges and yellow ligaments acting as primary factors in the reduction of canal amplitude in normal patients [11, 20]. The measurement is of considerable importance in the presence of suspected stenosis not detectable in the supine position, with the necessary considerations resulting from the therapeutic point of view.

The last investigated parameter, interspinous distance, showed a significant change in the passage from supine to upright position in both acute and chronic patients with instability. In the supine position, we could only identify indirect radiological signs of instability (degenerative disc, facet joints, ligament disease) and a few misalignments [21]. MRI in the standing position can detect changes in intersegmental motion and correlate it with symptoms. Instability can be considered part of the physiological degeneration of the lumbar spine and is divided into three phases: Initially, there is a change in movement of the complex consisting of the disc, some adjacent ligaments, and facet joints; signs of degeneration are minimal [21]. At this stage, there is movement dysfunction that may still not be appreciated in the upright MRI. In the next stage, the so-called instability phase, signs of degeneration are more appreciable, resulting in hypermobility of some spinal segments in comparison with supine MRI. In this phase, the excessive movement can lead to a higher degree of stenosis of the foramen and recesses, which may correlate with increased symptomatology. The disc below the affected level can show signs of degeneration and increased movement [22]. Progression of the degenerative phase leads to the appearance of osteophytes, with resultant restabilization and reduction in movement (step 3); this phase of restabilization is difficult to interpret without the aid of a dynamic study of the spine [23, 24]. In the beginning, degenerative or isthmic spondylolisthesis may appear stable without significant change in angular rotation or horizontal translation. As the degree of degeneration increases, it can be increasingly appreciated [22].

Limitations of this study are mainly due to two factors: in the upright position, patients with acute low back pain may find it difficult to maintain the immobility necessary for the duration of the imaging acquisition with a duration of at least 4 min for each sequence. A second and equally important negative factor is the difficulty sometimes encountered in evaluating the most lateral areas of the spine, such as foramen and lateral recesses.

In conclusion, supine MRI remains the technique of choice for detecting degenerative disc disease associated with acute and chronic low back pain. However, in about one of three cases, conventional MRI performed in the supine position is unable to answer the clinical question [2]; in these cases, or if it is necessary to assess more accurately the degree of spinal instability, particularly if surgical therapy is scheduled, the upright MRI performed dedicated equipment can be a complementary investigation to traditional MRI survey.
